# Association of mixed polycyclic aromatic hydrocarbons exposure with cardiovascular disease and the mediating role of inflammatory indices in US adults

**DOI:** 10.1265/ehpm.24-00091

**Published:** 2024-12-10

**Authors:** Tingwei Du, Xiaoli Shen, Runqing Zhan

**Affiliations:** 1 Department of Epidemiology and Health Statistics, School of Public Health, Qingdao University, Qingdao, 266071, China; 2 Qingdao Haici Hospital, No. 4 Renmin Rd, Qingdao, 266000, China

**Keywords:** Cardiovascular diseases, Polycyclic aromatic hydrocarbons, Inflammation indices, Mixed exposure, Bayesian kernel machine regression, Mediation effect, NHANES

## Abstract

**Background:**

Cardiovascular disease (CVD) is the leading cause of death worldwide. Few studies have investigated the effects of mixed polycyclic aromatic hydrocarbon (PAH) exposure on CVD prevalence. We aimed to evaluate the association between mixed PAHs exposure and CVD and determine the extent to which these links are mediated by inflammatory indices.

**Methods:**

We used National Health and Nutrition Examination Survey (NHANES) data from 2003 to 2016. Adults with a diagnosis of CVD and seven monohydroxylated PAH metabolites (OH-PAHs) in their urine samples were included. Multivariate logistic regression and Bayesian kernel machine regression (BKMR) models were used to estimate the association between single and mixed PAHs exposure and CVD. Mediation analysis was used to evaluate the mediating effect of inflammatory indices on the association between PAHs mixtures and CVD.

**Results:**

Here, 9136 individuals were included and 10.5% had CVD. Multivariate logistic regression analysis with all the OH-PAHs included that 2-hydroxyfluorene was found positively associated with increased odds of CVD. The BKMR analysis revealed that the overall effect of the seven PAH mixtures was positively associated with CVD. The univariate exposure-response function showed that 2-hydroxyfluorene was positively associated with CVD. Moreover, mediation analysis demonstrated that neutrophil-to-lymphocyte ratio and systemic immune inflammation index mediated the association between PAHs and CVD.

**Conclusions:**

Our findings highlight the complexity of the association between mixed PAHs exposure and CVD. At the same time, our study provides insight into the potential mechanisms of inflammation as a mediator between exposure to PAH mixtures and CVD.

**Supplementary information:**

The online version contains supplementary material available at https://doi.org/10.1265/ehpm.24-00091.

## 1. Introduction

Cardiovascular diseases (CVD), a group of heart and blood vessel diseases represented mainly by coronary heart disease and cerebrovascular disease (stroke), are the leading causes of death worldwide [1, 2]. According to World Health Organization (WHO), global CVD deaths will increase from 17.5 million in 2012 to over 22 million by 2030 [3]. Genetics, tobacco, alcohol, and metabolic diseases, such as hypertension and dyslipidemia, are risk factors for CVD [4]. Recently, the influence of environmental contaminants on CVD is gaining popularity in research. Environmental pollution increases the prevalence of CVD [5].

Polycyclic aromatic hydrocarbons (PAHs) are endocrine-disrupting chemicals that originate from the incomplete combustion of organic substances, including tobacco, fossil fuels, wood, petroleum products, and food processing [6]. Airborne PAHs have half-lives of several days [7]. Nevertheless, owing to their high hydrophobicity, PAHs are readily adsorbed onto solid particles, potentially extending their half-lives and rendering them persistent environmental micropollutants [8]. PAHs permeate the body through the skin, lungs, and gastrointestinal tract, and subsequently undergo oxidation by cytochrome P450 enzymes to form several monohydroxylated polycyclic aromatic hydrocarbons (OH-PAHs), which are ultimately metabolized into glucuronates and sulfates [9]. Evaluating OH-PAHs in human urine, considering multiple routes of exposure, helps assess an individual’s recent exposure to PAHs by estimating the internal dose [10]. PAHs are commonly denoted as carcinogens, mutagens, and teratogens, and thus constitute a substantial contribution to human well-being [11]. PAH exposure is related to many chronic health problems such as childhood asthma, lung cancer, metabolic syndrome, hypertension, atherosclerosis, and CVD [12].

The National Priority List (NPL) issued by the U.S. Environmental Protection Agency (EPA) has recognized 1,408 hazardous waste sites, and PAHs have been found in at least 600 sites. Sixteen PAH components were listed in the inventory of pre-control pollutants by the EPA [13]. Several epidemiological studies have discerned a positive association between single PAH exposure and CVD in different populations [14–17]. A meta-analysis by Mirzababaei et al. reached a similar conclusion [18]. However, the studies mentioned above generally focused on the effect of exposure to a single PAH on CVD. Combustion of organic substances may produce complex mixtures of high molecular weight PAHs, including pyrene, benzo[a]pyrene(BaP), dibenzo(a,h)anthracene, dibenzo[a,l]pyrene, benzo[ghi]perylene, and others [19]. A case-control study showed that a variety of PAHs were simultaneously detected in the serum of asthmatic children [20]. The complex exposure pattern and interactions between mixture PAHs require a customized approach to assess their combined effects. Hence, Bobb et al. proposed a new modeling technique called Bayesian kernel machine regression (BKMR). BKMR is a high-dimensional exposure-response function allowing nonlinearity and/or interaction among the mixed chemicals [21].

Leukocytes and platelets participate in both local and systemic inflammation, and in the pathogenesis of CVD [22, 23]. Inflammatory indices based on complete blood counts, such as the platelet-to-lymphocyte ratio (PLR) and neutrophil-to-lymphocyte ratio (NLR), have been suggested as predictors of CVD [24]. The systemic immune-inflammation index (SII), calculated using platelet, neutrophil, and lymphocyte counts to simultaneously reflect the host’s inflammatory and immune statuses, was initially used to predict the prognosis of certain cancers. The SII has also been discovered to serve as a superior predictor of significant cardiovascular events [25]. Moreover, PAH exposure may induce systemic inflammation [26, 27]. However, whether inflammation plays a mediating role in the association between PAHs and CVD remains unclear.

Herein, we used the National Health and Nutrition Examination Survey (NHANES) 2003–2016 to investigate whether mixed PAHs exposure increased the odds of CVD, including congestive heart failure, coronary heart disease, angina, heart attack, and stroke by logistic regression and BKMR approach. We further evaluated the mediating effects of the inflammatory indices (PLR, NLR, and SII) on this relationship.

## 2. Methods

### 2.1 Study populations

NHANES comprises a series of surveys designed by the National Center for Health Statistics to continuously monitor the health status of the institutionalized population of the United States. NHANES collects data from representative American samples through interviews, examinations, and laboratory tests designed using multilevel probability sampling. All participants signed an informed consent form before participation, ensuring that the study met ethical standards and was approved by the Institutional Review Board of the Center for Disease Control and Prevention (CDC). The details are available on the official CDC website (https://wwwn.cdc.gov/nchs/nhanes/).

**Fig. 1:**
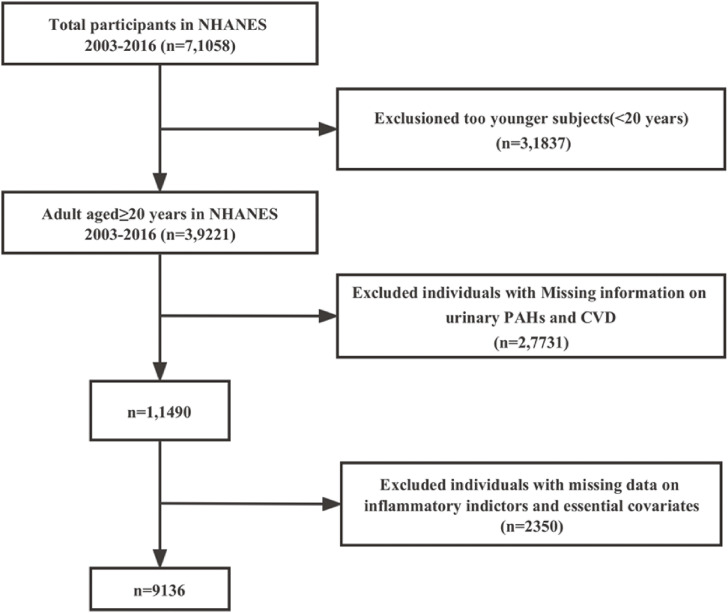
The Flow chart for included participants.

In the current study, we merged seven (2003–04, 2005–06, 2007–08, 2009–10, 2011–12, 2013–14 and 2015–16) data cycles from the NHANES. Overall, 7,1058 participants took part in the study over seven cycles. We excluded individuals 1) aged <20 years (N = 3,1837), 2) without CVD outcomes (N = 92), 3) with missing urinary OH-PAHs data (N = 2,7731), and 4) with missing inflammatory indices and covariates were eliminated from the data. Finally, 9136 participants with complete urinary OH-PAHs, including 8176 non-CVD and 960 CVD were enrolled, as shown in Fig. 1.

### 2.2 Measurement of urinary levels of OH-PAHs

Urine specimens were collected from the subjects, labeled, and stored at or below −20 °C. In the NHANES 2003–16, seven OH-PAHs were measured, including 1-hydroxynaphthalene (1-OHNAP), 2-hydroxynaphthalene (2-OHNAP), 2-hydroxyfluorene (2-OHFLU), 3-hydroxyfluorene (3-OHFLU), 1-hydroxyphenanthrene (1-OHPHE), 1-hydroxypyrene (1-OHPYR) and 2 & 3-hydroxyphenanthrene (2 & 3-OHPHE). During the 2003–2012 NHANES survey cycle, OH-PAHs in urine were measured using gas chromatography-tandem mass spectrometry (GC-MS/MS). High-performance liquid chromatography-tandem mass spectrometry (HPLC-MS/MS) was used in the 2013–2016 survey cycles. Concentrations of OH-PAHs below the lower limits of detection (LOD) were replaced with values equivalent to the LOD divided by the $\sqrt{2}$ . To account for the variance in urine sample volume, the exposure variables were adjusted for urinary creatinine levels in all analyses. Each OH-PAH (ng/L) was divided by urinary creatinine (mg/dl) and multiplied by 0.01 to yield nanograms of OH-PAHs per gram of creatinine (ng/g).

### 2.3 Measurement of inflammatory indices

Inflammatory indices were calculated based on complete blood counts. Using a Beckman Coulter DxH 800 instrument to measure complete blood counts on the blood samples at the NHANES Mobile Examination Center. Platelets, neutrophils, and lymphocytes were counted in 1000 cells/µl. To calculate the PLR, NLR and SII, the formulas were described below:

PLR = (total lymphocyte count)/(total platelet count); NLR = (absolute neutrophil count)/(total lymphocyte count); and SII = (absolute neutrophil count × total platelet count)/(total lymphocyte count).

### 2.4 Assessment of cardiovascular disease

CVD outcomes were self-reported during personal interviews using a validated medical condition questionnaire. Participants were asked the following question: “Has a doctor or other health professional ever told you that you have chronic heart failure (CHF)/coronary heart disease (CHD)/angina/heart attack/stroke?” (There were five separate questions with the same wording). If a participant answered “yes” to any of the above questions, he or she was considered a patient with CVD. Therefore, the outcome was converted into a dichotomous variable. Participants who answered all questions with “I don’t know” were excluded.

### 2.5 Covariates

Covariates that influence the association between PAH concentration and CVD were obtained through direct interviews and medical center examinations; they included sociodemographic information such as age (continuous variable), sex (male/female), race (Mexican American, other Hispanic, non-Hispanic white, non-Hispanic black, and other race—including multiracial), education level (less than high school, high school or equivalent, college or above), marital status (married/living with partner, widowed/divorced/separated, and never married), the ratio of household income to poverty (<1, 1–2, >2 [PLR]). Hypertension (yes, no), alcohol consumption (yes, no), smoking status (yes, no), and family history of CVD (yes, no) were collected during the family interview utilizing a standardized questionnaire. Body mass index (kg/m^2^), and serum cotinine concentration (<1, ≥1 ng/mL), were collected by physical measurements, or laboratory testing.

Body mass index (BMI) was calculated in kg/m^2^. Using BMI cutoff points, the participants were divided into three groups: non-obese (<25 kg/m^2^), normal weight (25–30 kg/m^2^), and obese (>30 kg/m^2^) [28]. Alcohol consumption was defined as consumption of at least 12 alcoholic drinks per year. Smoking status was defined as having at least 100 cigarettes in one’s lifetime. Participants who met any of the following criteria were defined as having hypertension: 1) systolic blood pressure (SBP) ≥ 140 mmHg, a mean diastolic blood pressure (DBP) ≥ 90 mmHg; 2) “Did a doctor tell you that you have high blood pressure?” if participants replied “yes”, they were categorized as having hypertension; or 3) use of antihypertensive medication [29].

### 2.6 Statistical analysis

Continuous variables were expressed as mean and standard deviation (SD) if they had a normal distribution; otherwise, medians and interquartile ranges (IQRs) were used. Student’s t-test (normally distributed continuous variables), and Mann-Whitney U test (non-normally distributed continuous variables) were used to compare group differences. Categorical variables are expressed as frequencies and percentages, and Chi-square tests were performed to compare the distribution differences. Spearman’s correlation coefficient was used to depict the associations among all OH-PAHs. Statistical significance was defined as *p*-value < 0.05.

All OH-PAHs were modeled as continuous (Ln-transformed) and categorically defined by quartiles (Q1, Q2, Q3, and Q4). All models were adjusted for age, sex, race, educational level PLR, marital status, alcohol consumption, smoking status, BMI, hypertension, and family history of CVD. Logistic regression analysis was performed to evaluate the association between PAHs and the prevalence of CVD using odds ratios (OR) and 95% confidence intervals (CIs). The BKMR model was then used to assess the overall effect of joint exposure to seven PAHs. This is an algorithm implementing Markov chain Monte Carlo (MCMC) to estimate the overall association of mixed chemical compounds with outcomes through a Gaussian kernel iterative regression of high-dimensional exposure-response functions. According to Spearman’s rank correlation values, we divided the 7 OH-PAHs into five groups (Group 1: 1-OHNAP; Group 2: 2-OHNAP; Group 3: 3-OHFLU and 2-OHFLU; Group 4: 1-OHPHE and 2 & 3-OHPHE; Group 5: 1-OHPYR). We used a hierarchical variable selection method to estimate the posterior inclusion probabilities, including group posterior probabilities of inclusion (groupPIP) and conditional posterior probabilities of inclusion (condPIP). The former is the probability that a mixed group will be included in the final model. The latter is based on the former, which reflects the likelihood that particular chemicals in a group will be included in the model. In this study, the joint effect was estimated by comparing all OH-PAHs at the 60th percentile level or above with those exposed at the 50th percentile level. Then, we estimated the exposure-response relationship by fixing all other PAHs to their median exposure levels. Moreover, we assessed how the change of an individual PAH between the 25th and 75th percentile contributes to the outcome when other PAHs were set at the 25th, 50th, and 75th percentile. The model was fitted with 10,000 iterations. Finally, to explore whether inflammatory indices mediate the association between PAHs levels and the prevalence of CVD, we performed a mediation analysis. The exposure-response relationship between PAHs (X) and CVD (Y) is mediated by inflammation parameters (M). The total effects (TE) of PAHs were split into direct effects (DE) and indirect effects (IE) on CVD. The a path represents the exposure-mediator effect (X and M), and the b path represents the mediator-outcome effect (M and Y), as shown in Fig. S1. Based on the method suggested by Rijnhart, an indirect effect is considered to have occurred when there is a statistically significant product of the a and b coefficients (ab). In addition, the proportion mediated was obtained by calculating the ratio of the indirect effect over the total effect, thus quantifying the magnitude of the mediation effect [30]. The significance of the mediation effect was assessed using Bootstrap sampling (times = 1000).

We organized the data using Stata (version 16.0) software. For statistical analysis, R software (version 4.3.1) was used. BKMR analysis and Mediation analysis were performed using the bkmr package and mediation package in R software, respectively. Statistical significance was defined as *p*-value < 0.05.

## 3. Results

### 3.1 Population characteristics

Overall, 9136 participants were included in the analysis. CVD prevalence was 10.5% (n = 960). The average age of the non-CVD and CVD populations was 46.9 ± 17.3 and 65.6 ± 13.2 years old, and the proportion of males was 59.8% and 48.8%, respectively. The median NLR, PLR, and SII for the non-CVD population were 119.5 (92.0–154.3), 507.2 (353.3–718.0), and 2.2 (1.7–3.0), respectively. For the CVD population, the median values of NLR, PLR, and SII were 119.2 (94.3–150.0), 472.0 (335.8–666.5), and 1.9 (1.4–2.6), respectively. There were significant differences between the CVD and non-CVD groups in terms of age, sex, race, educational level, marital status, PLR, smoking status, BMI, hypertension, and family history of CVD (*p* < 0.05). Compared to participants without CVD, those with CVD were older, male, and more likely to be Mexican Americans, with lower levels of education, obesity, former smoking, higher prevalence of hypertension, and a family history of CVD (Table 1).

**Table 1:** Participant characteristics (N = 9136) in NHANES, (2003–2016).

**Characteristics**	**CVD (n = 960)**	**Non–CVD (n = 8176)**	***p* value**
**Age (year), mean ± SD**	65.6 ± 13.2	46.9 ± 17.3	<0.001
**Sex, n (%)**			<0.001
**Male**	574 (59.8)	3963 (48.8)	
**Female**	386 (40.2)	4213 (51.5)	
**Race, n (%)**			<0.001
**Mexican American**	103 (7.0)	1364 (93.0)	
**Other Hispanic**	68 (9.8)	706 (91.2)	
**Non–Hispanic White**	563 (13.3)	3668 (86.7)	
**Non–Hispanic Black**	183 (10.0)	1655 (90.0)	
**Other race, including multi–racial**	43 (5.2)	783 (94.8)	
**Education level, n (%)**			<0.001
**Less than high school**	338 (15.2)	1884 (84.8)	
**High school or equivalent**	227 (10.6)	1905 (89.4)	
**College or above**	395 (8.3)	4387 (91.7)	
**Marital status, n (%)**			<0.001
**Married/Living with partner**	539 (9.8)	4986 (90.2)	
**Widowed/Divorced/Separated**	358 (10.6)	1598 (89.4)	
**Never married**	63 (5.8)	1592 (96.2)	
**PIR, n (%)**			<0.001
**<1**	225 (11.9)	1662 (88.1)	
**1–2**	321 (13.3)	2095 (86.7)	
**>2**	414 (9.6)	4419 (91.4)	
**Drinking status, n (%)**			0.205
**Yes**	280 (11.2)	2227 (88.8)	
**No**	680 (10.3)	5949 (89.7)	
**Smoking status, n (%)**			<0.001
**Yes**	248 (7.6)	3001 (92.4)	
**No**	712 (12.1)	5175 (87.9)	
**BMI category, n (%)**			<0.001
**Non–obese**	219 (9.2)	2461 (91.8)	
**Normal weight**	312 (10.2)	2757 (89.8)	
**Obese**	429 (12.7)	2958 (87.3)	
**Hypertension, n (%)**			<0.001
**Yes**	694 (19.2)	2918 (80.8)	
**NO**	266 (4.9)	5258 (95.1)	
**Family history of CVD, n (%)**			<0.001
**Yes**	245 (17.9)	1121 (82.1)	
**No**	715 (9.2)	7055 (90.8)	
**serum cotinine (ng/mL)**			
**<1**	5663 (89.6)	653 (10.4)	0.519
**≥1**	2442 (89.5)	293 (10.5)	
**Platelet count, 10^9/L, median (IQR)**	225.0 (186.0–27.0)	244.0 (206.0–288.0)	<0.001
**Lymphocyte count, 10^9/L, median (IQR)**	1.9 (1.5–2.4)	2.1 (1.7–2.6)	<0.001
**Neutrophil count, 10^9/L, median (IQR)**	4.3 (3.4–5.5)	4.0 (3.1–5.2)	<0.001
**NLR**	119.5 (92.0–154.3)	119.2 (94.3–150.0)	0.901
**PLR**	507.2 (353.3–718.0)	472.0 (335.8–666.5)	<0.001
**SII**	2.2 (1.7–3.0)	1.9 (1.4–2.6)	<0.001

Note: Categorical variables are presented as n (%), *p* value are generated by Chi-Square tests. Continuous variables are presented as mean (sd) or median (IQR). *p* value are generated by Student’s t-test or Mann-Whitney U test.

BMI = body mass index; CVD = cardiovascular disease; Non-CVD = Non cardiovascular disease; NHANES, National Health and Nutrition Examination Survey; NLR, neutrophil–to–lymphocyte ratio; PIR = poverty–income ratio; PLR, platelet–to–lymphocyte ratio, SII, systemic immunity–inflammation index.

### 3.2 Distribution of urinary OH-PAHs levels and their correlation

Table S1 shows the detection frequency and exposure distribution of the seven OH-PAHs concentrations among the study participants. The detection frequency of all the OH-PAHs substances was >90%. The highest and lowest concentrations of urinary OH-PAHs were 1-OHNAP and 2-OHNAP, with geometric means of 3442.64 ng/L and 2861.41 ng/L, respectively. The correlation coefficients of the seven urinary OH-PAHs levels were determined using Spearman’s rank correlation analysis. All OH-PAHs showed positive correlations from 0.32 to 0.95. Additionally, 2-OHFLU levels were highly correlated with 1-OHFLU (*r* = 0.95, *p* < 0.001). There was a strong correlation between 1-OHPHE and 2 & 3-OHPHE (*r* = 0.83, *p* < 0.001) (Fig. S2).

### 3.3 Associations between urinary OH-PAH levels and prevalence of CVD

#### 3.3.1 Single urinary OH-PAHs and CVD risk: logistic regression

Table S2 presents the results of the multivariate logistic regression analysis. After adjusting for all covariates, the ORs (95% CIs) of CVD for 1-OHNAP, 2-OHNAP, 3-OHFLU, and 2-OHFLU were 1.07 (1.02–1.13), 1.13 (1.05–1.22), 1.15 (1.07–1.22) and 1.20 (1.11–1.29), respectively. Compared with the lowest quartile, we found that the 4th quartile of 1-OHNAP (OR: 1.32, 95% CI: 1.06–1.64), 2-OHNAP (OR: 1.45, 95% CI: 1.69–1.79), 3-OHFLU (OR: 1.43, 95% CI: 1.14–1.78), 2-OHFLU (OR: 1.56, 95% CI: 1.24–1.96), and 1-OHPYR (OR: 1.33, 95% CI: 1.07–1.65) were positively associated with the odds of CVD.

We fitted a multivariate logistic regression including all OH-PAHs to adjust for the confounding effects of other OH-PAHs. Only 2-OHFLU was significantly correlated with an elevated prevalence of CVD (Table 2). Compared to the lowest quartile, participants in the 2nd (OR: 1.56, 95% CI: 1.19–2.03), 3rd quartiles (OR: 1.97, 95% CI: 1.39–2.81), 4th quartile (OR: 1.32, 95% CI: 1.06–3.07) of 2-OHFLU had significantly increased odds of CVD. However, no association was observed between the remaining components and the prevalence of CVD after adjusting for covariates. Moreover, inverse probability weighted analysis was performed to exclude potential selection bias. The results were consistent with our main analysis (Table S3).

**Table 2:** Association between OH–PAHs and CVD with all the OH–PAHs included (N = 9136), NHANES (2003–2016).

**OH–PAHs**	**Quartile 1**	**Quartile 2**		**Quartile 3**		**Quartile 4**		**Total**	

		**OR (95% CI)**	***p* value**	**OR (95% CI)**	***p* value**	**OR (95% CI)**	***p* value**	**OR (95% CI)**	***p* value**
**1–OHNAP**	Ref.	1.00 (0.79–1.26)	0.998	0.87 (0.68–1.12)	0.282	1.03 (0.78–1.37)	0.808	1.02 (0.95–1.08)	0.590
**2–OHNAP**	Ref.	0.93 (0.75–1.16)	0.529	1.08 (0.86–1.37)	0.502	1.20 (0.91–1.58)	0.187	1.01 (0.91–1.12)	0.847
**3–OHFLU**	Ref.	0.83 (0.64–1.08)	0.172	0.72 (0.52–1.02)	0.064	0.95 (0.56–1.60)	0.854	0.89 (0.72–1.11)	0.309
**2–OHFLU**	Ref.	**1.56 (1.19–2.03)**	**0.001**	**1.97 (1.39–2.81)**	**0.000**	**1.32 (1.06–3.07)**	**0.029**	**1.58 (1.22–2.05)**	**0.000**
**1–OHPHE**	Ref.	0.80 (0.62–1.02)	0.075	0.82 (0.62–1.09)	0.170	0.77 (0.55–1.09)	0.142	0.84 (0.69–1.02)	0.075
**1–OHPYR**	Ref.	0.89 (0.71–1.11)	0.302	0.87 (0.68–1.12)	0.293	1.19 (0.88–1.62)	0.252	1.03 (0.90–1.17)	0.683
**2&3–OHPHE**	Ref.	0.83 (0.64–1.07)	0.156	0.82 (0.60–1.12)	0.218	0.77 (0.52–1.15)	0.207	0.85 (0.68–1.05)	0.135

Note: Model was adjusted for age, sex, race, education level, marital status, the ratio of household income to poverty (PLR), alcohol consumption, smoking status, BMI, hypertension, and family history of CVD, All OH–PAHs were ln-transformed, Quartiles were based on all the participants in our study, Total means continuous chemical variable.

CI = Confidence Interval, NHANES = National Health and Nutrition Examination Survey, OR = Odds Ratios; OH-PAHs, urinary metabolites of Polycyclic aromatic hydrocarbons; Ref. = Reference; 1–OHNAP = urinary metabolites of 1–Hydroxynaphthalene; 2–OHNAP = urinary metabolites of 2–Hydroxynaphthalene; 3–OHFLU = urinary metabolites of 3–Hydroxyfluorene; 2–OHFLU = urinary metabolites of 2–Hydroxyfluorene; 1–OHPHE = urinary metabolites of 1–Hydroxyphenanthrene; 1–OHPYR = urinary metabolites of 1–Hydroxypyrene; 2&3–OHPHE = urinary metabolites of 2&3–Hydroxyphenanthrene.

#### 3.3.2 Evaluate the combined association between PAHs exposure and CVD risk: BKMR model

**Fig. 2:**
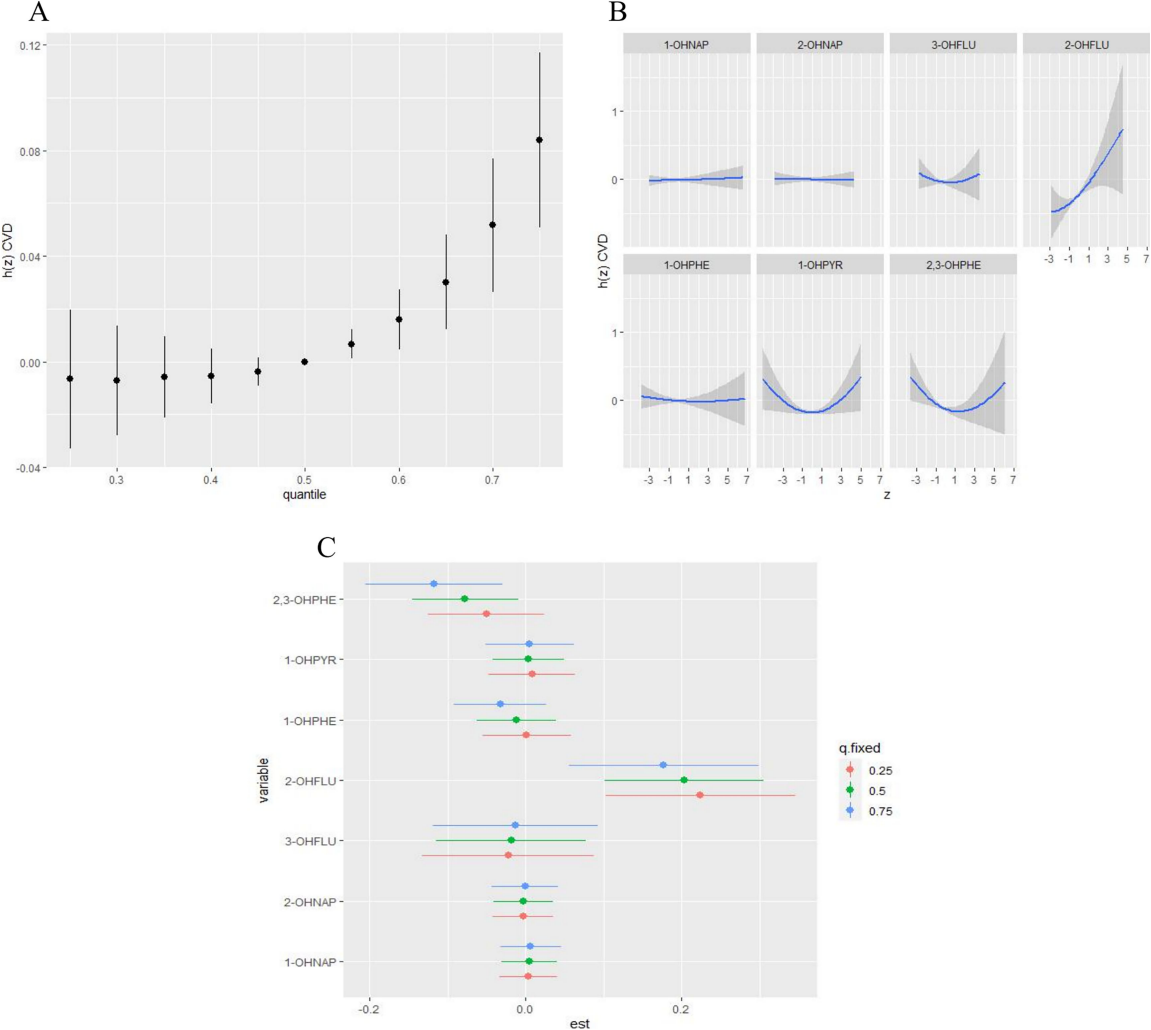
Association between PAHs exposure and CVD risk by hierarchical BKMR model. h(z) can be interpreted as the relationship between PAHs and CVD risk. The model age, sex, race, education level, marital status, PLR, alcohol consumption, smoking status, BMI, hypertension, and family history of CVD. (A) Joint effects (95% CI) of mixed PAHs exposure on CVD estimated by comparing when all dioxins at particular percentiles (from 0.25 to 0.75, increments of 0.05) with when all the PAHs at their 50th percentile. (B) Univariate exposure–response functions (95% CI) between each PAHs concentrations and CVD with fixing the concentrations of other PAHs at their 50th percentile. (C) The relationship of individual OH–PAHs with CVD when an individual OH–PAH exposure was at its 75th percentile as compared to its 25th percentile, when all of the other OH–PAHs were fixed at a specific exposure percentile (25th, 50th, or 75th, respectively).

We fitted the BKMR model to analyze the combined effect of PAHs exposure on CVD. When all OH-PAHs were at their 55th percentile or above, compared to their 50th percentile, a significant positive tendency was found between mixed OH-PAHs and CVD (Fig. 2(A)). The trends in the exposure-response functions of the seven OH-PAHs were further assessed, as shown in Fig. 2(B). When all other OH-PAHs were taken as median values, 2-OHFLU showed a positive association with the odds of CVD, while 1-OHNAP, 2-OHNAP, and 1-OHPHE showed a slightly positive relationship. Finally, we estimated the change in the risk of CVD associated with an IQR increase in the level of a single PAH, while the other OH-PAHs are fixed at 25th, 50th, or 75th percentile levels, respectively (Fig. 2(C)). 2-OHFLU had a positive effect on the CVD odds when the remaining OH-PAHs were set at the 25th, 50th, and 75th percentiles.

The contributions of the seven urinary OH-PAHs to the overall effect were further explored. We divided the seven urinary OH-PAHs into five groups based on their correlations with each other. The groupPIP and condPIP values for each OH-PAH are summarized in Table S4. The PIPs of the BKMR model indicated that the 2 & 3-OHFLU group (Group 3) had the highest groupPIP, with 2-OHFLU driving the main effect, followed by 3-OHFLU (groupPIP: 1.00, 2-OHFLU condPIP: 0.75, 3-OHFLU condPIP: 0.25).

### 3.4 Inflammatory indices mediated the association between urinary OH-PAHs levels and CVD

**Fig. 3:**
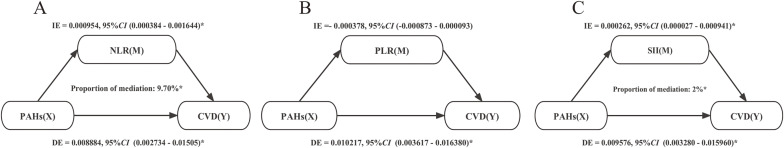
Estimated proportion of the association between urinary PAHs and CVD mediated by PLR, NLR, SII. The model age, sex, race, education level, marital status, PLR, alcohol consumption, smoking status, BMI, hypertension, and family history of CVD. IE, estimate of the indirect effect; DE, estimate of the direct effect. PAHs, polycyclic aromatic hydrocarbons; PLR, platelet–to–lymphocyte ratio; NLR, neutrophil–to–lymphocyte ratio; SII, systemic immunity–inflammation index; CVD, cardiovascular diseases.

Table S5 shows the association between urinary OH-PAHs and inflammatory indices (PLR, NLR, and SII). The association between inflammatory indices (PLR, NLR, and SII) and CVD was shown in Table S6. Then, we performed a mediation analysis to identify whether inflammatory indices mediated the impact of PAHs on the prevalence of CVD. We found that a mixture of PAHs may contribute to the development of CVD by affecting NLR and SII (Fig. 3). As shown in Fig. 3A, NLR-mediated efficacy accounted for 9.7% of the association between PAHs and the prevalence CVD (IE = 0.000954, 95%CI: 0.000384–0.001644; DE = 0.0089, 95% CI: 0.002734–0.015050), and as presented in Fig. 3C, SII-mediated efficacy accounted for 2.0% (IE = 0.000262, 95%CI: 0.000027–0.000941; DE = 0.009676, 95% CI: 0.003280–0.015960).

## 4. Discussion

To our knowledge, this is the first study to assess the combined effects of exposure to seven PAHs on CVD. Our main findings (1) In the BKMR analysis, we found that the overall effect of PAHs mixture was positively correlated with the prevalence of CVD. The univariate exposure-response function showed that 2-OHFLU was positively associated with CVD risk. (2) When all OH-PAHs were included in the multivariate logistic regression analysis simultaneously, 2-OHFLU was positively associated with CVD. (3) The NLR and SII had a mediating effect on the association between mixed PAHs exposure and CVD risk. NLR and SII mediated 9.7% and 2.0% of the association, respectively.

Several epidemiological studies using multi-chemical linear regression analyses have found a connection between single PAH and CVD [14–17]. A study from the Wuhan-Zhuhai cohort in China demonstrated that urinary levels of 2-hydroxyfluoren, 9-hydroxyfluoren, and 1-hydroxyphenanthrene increased the risk of atherosclerotic cardiovascular disease [14]. Hajir et al. found that 1-pyrenol was a risk factor for obstructive coronary artery disease in Lebanon [15]. Alshaarawy et al. reported a positive association between eight urinary PAH biomarkers and CVD [16]. Mallah et al. showed that 1-OHNAP, 2-OHNAP, 3-OHFLU, 2-OHFLU, 1-OHPYR, and 1-OHNAP increased the risk of CVD [17]. Usually, a single PAH is included in these analyses, making their results easy to understand. The highly correlated, nonlinear, and non-additive relationships between mixture components and health cannot be resolved using a multi-chemical linear model or structural equation model. Disregarding the joint effects of mixture components can lead to false-positive or false-negative results [31]. In our study, we showed that when all OH-PAHs were at their 55th percentile or above, compared to their 50th percentile, a significant positive tendency was found between mixed OH-PAHs and CVD. Moreover, 2-OHFLU was the most important contributor to CVD (PIP = 0.75) in the BKMR model and positively associated with the odds of CVD in the multivariate logistic regression analysis.

Compared with epidemiological studies, laboratory investigations help better observe the effects of PAHs on cardiac function. Pheorene exposure has been associated with increased susceptibility to arrhythmia in mammals [32]. Exposure to PAHs can result in a reduction of heart mass by downregulating the TGF-β1/Smad3 signaling pathway [33]. PAHs and their metabolites may initiate vessel injury leading to accelerated atherosclerosis [34]. There are multiple biological mechanisms underlying PAH exposure-induced increase in CVD risk. First, PAHs enter the human body and are metabolized into active semiquinones by cytochrome P450 enzymes to produce reactive oxygen species (ROS) in several organs. Excessive ROS production damages endothelial cells, especially in terminal arteries, and causes endothelial cell dysfunction [35, 36]. Second, mixed PAH exposure is positively correlated with thyroid hormone levels [37], and low thyroid function may adversely affect the development of atherosclerotic manifestations [38]. Third, PAHs may cause atherosclerosis by activating aromatic receptors and inflammatory cytokines [39].

PLR, NLR, and SII are innovative and predictive inflammatory indices that bind platelets, neutrophils, and lymphocytes, and serve as reliable indicators of thrombosis and inflammation [40]. Our findings are similar to those of previous studies showing that NLR and SII increase the odds of CVD [24, 41]. Serrano et al. showed that NLR was associated with an increased Coronary Artery Calcification score [24]. Zheng et al. observed a positive correlation between SII and heart failure prevalence [41]. Our results showed that a combination of urinary OH-PAHs was associated with increased NLR and SII. A similar finding was reported in that urinary OH-PAHs were associated with increased neutrophil count, NLR, and SII [27]. The role of inflammatory indices as mediators of the mechanisms linking PAHs to CVD remains unclear. Initially, the exposure to PAHs resulted in a significant amount of ROS. ROS directly inflicts oxidative damage to the cell membrane. Subsequently, oxidative stress acts as a danger signal, activating the inflammasome, which detects danger signals both inside and outside cells, thereby initiating inflammation. By activating the inflammasome and other inflammatory signaling pathways, these cytokines activate WBCs such as neutrophils and macrophages, facilitating the aggregation of inflammatory cells and enhancing inflammation [42, 43]. Subsequently, platelet-neutrophil interactions are involved in key processes linked to atherosclerosis, thrombosis, and ischemic stroke [44]. Here, we found that NLR and SII could mediate 9.7% and 2.0% of the associations between PAHs mixture exposure and the odds of CVD. Therefore, at the epidemiological level, PAH exposure may mediate CVD by activating the inflammatory pathways. We found that a combination of PAHs was associated with a reduced PLR. However, we did not find a mediating role of the PLR in the association between PAHs and CVD risk. Yuan et al. examined the nonlinearity of the relationship between urinary OH-PAHs and PLR in Wuhan participants using different bistatic angle-restricted cubic spline curves. However, no relationship was found between urine OH-PAHs and PLR in Zhuhai participants [45]. The results of previous studies have been inconsistent, and more animal studies are needed to determine the relationship between PLR and PAHs and whether PLR mediates PAHs and CVD risk.

There is no specific legislation addressing PAH abatement. The National Institute for Occupational Safety and Health (NIOSH) has established recommended occupational exposure limits for PAHs such as a recommended exposure limit of 0.1 mg/m^−3^ for coal tar pitch volatile agents for a 10-hour workday or 40-hour workweek [46]. The target annual average concentration for the PM10 fraction did not exceed 1 ng/m^−3^. However, recent findings indicate that many areas across Europe surpass this target [47].

This study has several advantages. First, the BKMR model was used to assess the combined effect of PAH exposure and CVD risk. The BKMR model fits the exposure-response function of the overall PAH exposure, as well as that of each PAH when the other PAHs were fixed at certain levels. The BKMR model may reveal nonlinear and non-additive correlations between PAHs. Second, we explored the mediating effect of inflammation on the association between PAHs and CVD at the epidemiological level. This study has some limitations. First, as a cross-sectional study, we could only observe associations, not establish causation. Second, the use of a self-reported questionnaire to define CVD may have introduced recall bias. Third, PAH exposure is closely related to occupational factors and was not considered in this study because of the lack of relevant occupational data in the NHANES. Fourth, urinary PAH metabolite levels were determined using measurements at a single time point. A single sample may not fully capture the long-term accumulation effects of PAH exposure or how the timing of exposure could affect CVD outcomes. Further follow-up studies are required to obtain more comprehensive data on PAH exposure and its long-term implications, to ascertain the long-term consequences of PAH exposure on CVD.

## 5. Conclusion

Logistic regression models, BKMR models, and mediation effect analysis indicated that mixed PAH exposure increases CVD prevalence, and this process is mediated through inflammation. Moreover, 2-hydroxyfluorene is the most important factor associated with CVD. Regulation of atmospheric PAHs may be an effective technique for lowering and preventing the risk of CVD.

## Abbreviations

BMI: Body mass index
CVD: Cardiovascular diseases
CHF: chronic heart failure
CIs: confidence intervals
CHD: coronary heart disease
DBP: diastolic blood pressure
DE: direct effects
GC-MS/MS: gas chromatography-tandem mass spectrometry
HPLC-MS/MS: High-performance liquid chromatography-tandem mass spectrometry
IE: indirect effects
IQRs: interquartile ranges
LLOD: lower limits of detection
MCMC: Markov chain Monte Carlo
OH-PAHs: monohydroxylated polycyclic aromatic hydrocarbons
NHANES: National Health and Nutrition Examination Survey
NLR: neutrophil-to-lymphocyte ratio
OR: odds ratios
PLR: platelet-to-lymphocyte ratio
PAHs: Polycyclic aromatic hydrocarbons
SD: standard deviation
SII: systemic immune-inflammation index
WHO: World Health Organization

## Supplementary information

**Additional file 1:** **Figure S1.** Path diagram of a single mediator model.

**Figure S2.** Spearman’s rank correlations between creatinine-corrected OH-PAH levels (ng/g creatinine).

**Table S1.** Distribution of urinary metabolites of Polycyclic aromatic hydrocarbons, NHANES 2003–2016, (n = 9136).

**Table S2.** Association of single urinary polycyclic aromatic hydrocarbons levels with CVD (N = 9136), NHANES (2003–2016).

**Table S3.** Association of urinary polycyclic aromatic hydrocarbons levels with CVD (N = 9136), NHANES (2003–2016).

**Table S4.** Posterior inclusion probabilities (PIPs) for group inclusion (groupPIP) and conditional inclusion (condPIP), using the Bayesian kernel machine regression (BKMR) model (N = 9136), NHANES (2003–2016).

**Table S5.** Multivariate analysis of the association of urinary mixed of PAHs and concentration changes (95% CI) in inflammatory indicators.

**Table S6.** Association of inflammatory indicators levels with CVD (N = 9136), NHANES (2003–2016).

## Declarations

### Availability of data and materials

The datasets can be found in online repositories. At: https://www.cdc.gov/nchs/nhanes/index.htm.
