# Supplementary figures and images for: Association of mixed polycyclic aromatic hydrocarbons exposure with cardiovascular disease and the mediating role of inflammatory indices in US adults
Source: Environ Health Prev Med. 2024 Dec 10;29:70. doi: 10.1265/ehpm.24-00091 (PMC11652969; doi:10.1265/ehpm.24-00091)

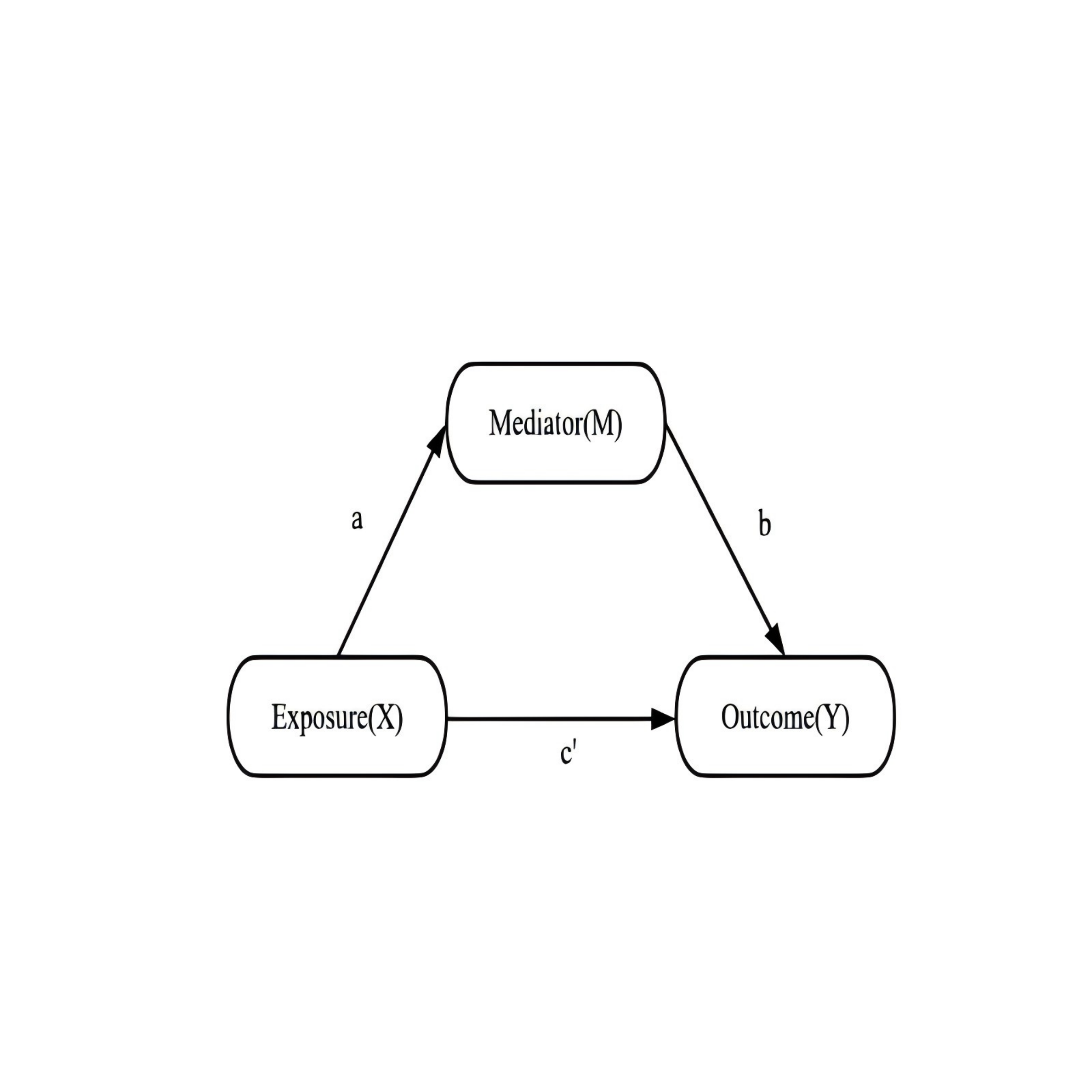

Supplement: Supplementary file 1 — Additional file 1: Figure S1. Path diagram of a single mediator model. [file ehpm-29-070-s001.tif]

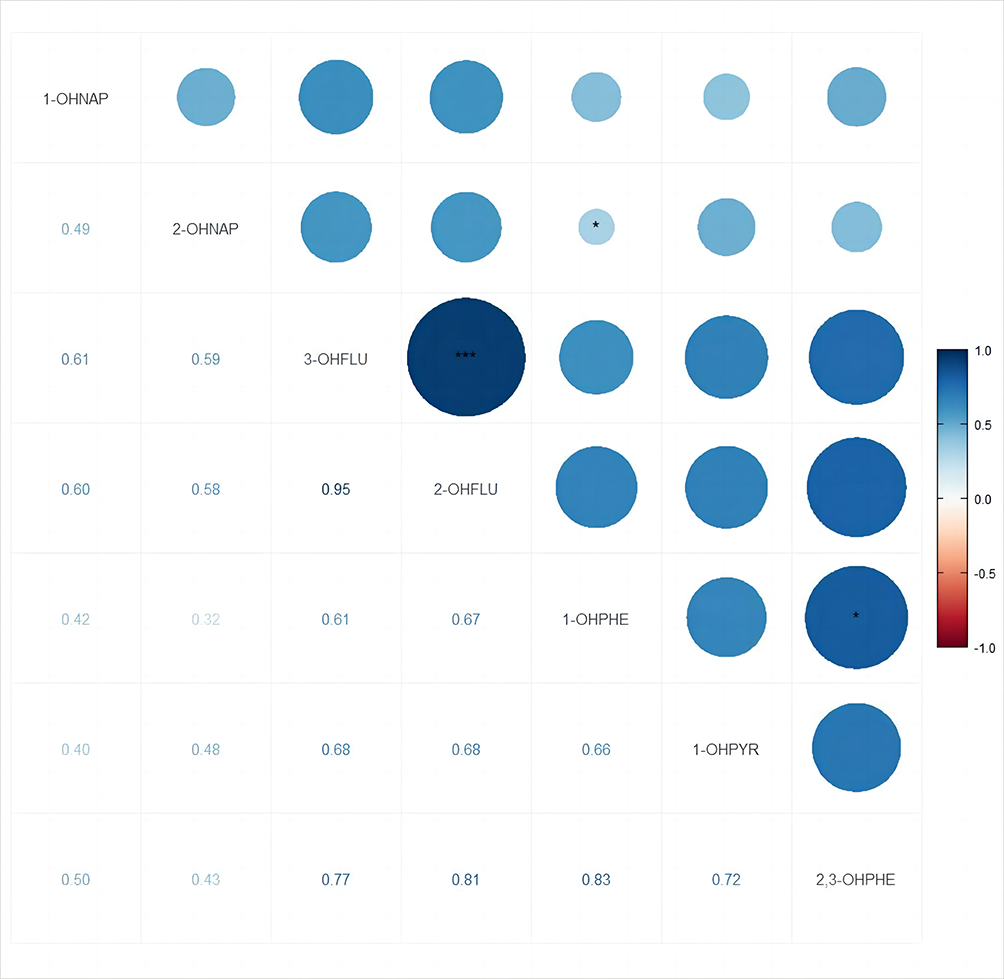

Supplement: Supplementary file 2 — Figure S2. Spearman’s rank correlations between creatinine-corrected OH-PAH levels (ng/g creatinine). [file ehpm-29-070-s002.tif]
