# Supplementary material for: Association of mixed polycyclic aromatic hydrocarbons exposure with cardiovascular disease and the mediating role of inflammatory indices in US adults
Source: Environ Health Prev Med. 2024 Dec 10;29:70. doi: 10.1265/ehpm.24-00091 (PMC11652969; doi:10.1265/ehpm.24-00091)
Supplement: Supplementary file 3 — Table S1. Distribution of urinary metabolites of Polycyclic aromatic hydrocarbons, NHANES 2003–2016, (n = 9136). [file ehpm-29-070-s003.docx]

| Table S1. Distribution of urinary metabolites of Polycyclic aromatic hydrocarbons, NHANES 2003–2016, (n = 9136). | | | | | | | | |
| --- | --- | --- | --- | --- | --- | --- | --- | --- |
| **OH–PAHs** | **detection frequency** | **GM (ng/L)** | **mean (ng/L)** | **Percentile** | | | | |
|  |  |  |  | **5th** | **25th** | **50th** | **75th** | **95th** |
| **1–OHNAP** | 93.3% | 3442.64 | 33783.14 | 245.00 | 765.70 | 1861.30 | 6638.00 | 32036.83 |
| **2–OHNAP** | 94.1% | 2861.41 | 8600.82 | 593.18 | 1868.00 | 4357.00 | 10299.07 | 29804.54 |
| **3–OHFLU** | 94.0% | 105.37 | 314.03 | 14.30 | 39.10 | 82.75 | 271.07 | 1474.30 |
| **2–OHFLU** | 94.3% | 269.45 | 615.73 | 39.00 | 110.10 | 230.00 | 591.67 | 2524.21 |
| **1–OHPHE** | 93.7% | 146.77 | 207.10 | 27.00 | 68.00 | 128.30 | 238.87 | 594.30 |
| **1–OHPYR** | 94.2% | 75.16 | 208.85 | 20.00 | 49.50 | 103.00 | 212.77 | 670.18 |
| **2&3–OHPHE** | 93.6% | 177.99 | 260.13 | 29.00 | 74.00 | 143.00 | 280.00 | 775.00 |
| GM, geometric mean; OH-PAHs = urinary metabolites of Polycyclic aromatic hydrocarbons; 1–OHNAP = urinary metabolites of 1–Hydroxynaphthalene; 2–OHNAP = urinary metabolites of 2–Hydroxynaphthalene; 3–OHFLU = urinary metabolites of 3–Hydroxyfluorene; 2–OHFLU = urinary metabolites of 2–Hydroxyfluorene; 1–OHPHE = urinary metabolites of 1–Hydroxyphenanthrene; 1–OHPYR = urinary metabolites of 1–Hydroxypyrene; 2&3–OHPHE = urinary metabolites of 2&3–Hydroxyphenanthrene. | | | | | | | | |
