# Supplementary material for: Association of mixed polycyclic aromatic hydrocarbons exposure with cardiovascular disease and the mediating role of inflammatory indices in US adults
Source: Environ Health Prev Med. 2024 Dec 10;29:70. doi: 10.1265/ehpm.24-00091 (PMC11652969; doi:10.1265/ehpm.24-00091)
Supplement: Supplementary file 4 — Table S2. Association of single urinary polycyclic aromatic hydrocarbons levels with CVD (N = 9136), NHANES (2003–2016). [file ehpm-29-070-s004.docx]

| Table S2. Association of single urinary polycyclic aromatic hydrocarbons levels with CVD (N = 9136), NHANES (2003–2016). | | | | | | | | | |
| --- | --- | --- | --- | --- | --- | --- | --- | --- | --- |
| **OH–PAHS** | **Quartile 1** | **Quartile 2** |  | **Quartile 3** |  | **Quartile 4** |  | **Total** |  |
|  |  | **OR (95% CI)** | ***p***  **value** | **OR (95% CI)** | ***p***  **value** | **OR (95% CI)** | ***p***  **value** | **OR (95% CI)** | ***p***  **value** |
| **1–OHNAP** | Ref. | 0.99 (0.79–1.24) | 0.940 | 0.93 (0.74–1.17) | 0.544 | **1.32 (1.06–1.64)** | **0.015** | **1.07 1.02–1.13)** | **0.005** |
| **2–OHNAP** | Ref. | 0.93 (0.75–1.14) | 0.468 | 1.13 (0.91–1.40) | 0.273 | **1.45 (1.69–1.79)** | **0.001** | **1.13 (1.05–1.22)** | **0.001** |
| **3–OHFLU** | Ref. | 0.94 (0.76–1.16) | 0.572 | 0.93 (0.75–1.15) | 0.511 | **1.43 (1.14–1.78)** | **0.002** | **1.15 (1.07–1.22)** | **0.000** |
| **2–OHFLU** | Ref. | 1.15 (0.93–1.43) | 0.188 | 1.19 (0.96–1.47) | 0.109 | **1.56 (1.24–1.96)** | **0.000** | **1.20 (1.11–1.29)** | **0.000** |
| **1–OHPHE** | Ref. | 0.83 (0.67–1.03) | 0.098 | 0.91 (0.73–1.13) | 0.390 | 1.03 (0.83–1.27) | 0.776 | 1.02 (0.93–1.13) | 0.642 |
| **1–OHPYR** | Ref. | 0.87 (0.71–1.07) | 0.184 | 0.88 (0.71–1.08) | 0.225 | **1.33 (1.07–1.65)** | **0.010** | 1.08 (0.99–1.17) | 0.058 |
| **2&3–OHPHE** | Ref. | 0.86 (0.69–1.06) | 0.164 | 0.92 (0.74–1.14) | 0.435 | 1.12 (0.91–1.38) | 0.275 | 1.07 (0.97–1.17) | 0.166 |
| Note: Model was adjusted for age, sex, race, education level, marital status, the ratio of household income to poverty (PLR), alcohol consumption, smoking status, BMI, hypertension, and family history of CVD. All OH–PAHs were ln-transformed, Quartiles were based on all the participants in our study, Total means continuous chemical variable.  CI = Confidence Interval, NHANES = National Health and Nutrition Examination Survey, OR = Odds Ratios; OH-PAHs = urinary metabolites of Polycyclic aromatic hydrocarbons; Ref. = Reference; 1–OHNAP = urinary metabolites of 1–Hydroxynaphthalene; 2–OHNAP = urinary metabolites of 2–Hydroxynaphthalene; 3–OHFLU = urinary metabolites of 3–Hydroxyfluorene; 2–OHFLU = urinary metabolites of 2–Hydroxyfluorene; 1–OHPHE = urinary metabolites of 1–Hydroxyphenanthrene; 1–OHPYR = urinary metabolites of 1–Hydroxypyrene; 2&3–OHPHE = urinary metabolites of 2&3–Hydroxyphenanthrene. | | | | | | | | | |
