# Supplementary material for: Association of mixed polycyclic aromatic hydrocarbons exposure with cardiovascular disease and the mediating role of inflammatory indices in US adults
Source: Environ Health Prev Med. 2024 Dec 10;29:70. doi: 10.1265/ehpm.24-00091 (PMC11652969; doi:10.1265/ehpm.24-00091)
Supplement: Supplementary file 6 — Table S4. Posterior inclusion probabilities (PIPs) for group inclusion (groupPIP) and conditional inclusion (condPIP), using the Bayesian kernel machine regression (BKMR) model (N = 9136), NHANES (2003–2016). [file ehpm-29-070-s006.docx]

| Table S4. Posterior inclusion probabilities (PIPs) for group inclusion (groupPIP) and conditional inclusion (condPIP), using the Bayesian kernel machine regression (BKMR) model (N = 9136), NHANES (2003–2016). | | | |
| --- | --- | --- | --- |
| **variable** | **group** | **groupPIP** | **condPIP** |
| **1–OHNAP** | 1 | 0.0104 | 1.0000 |
| **2–OHNAP** | 2 | 0.0098 | 1.0000 |
| **3–OHFLU** | 3 | 1.0000 | 0.2476 |
| **2–OHFLU** | 3 | 1.0000 | 0.7524 |
| **1–OHPHE** | 4 | 0.4586 | 0.1993 |
| **1–OHPYR** | 5 | 0.2884 | 1.0000 |
| **2&3–OHPHE** | 4 | 0.4586 | 0.8007 |
| Note: The result was adjusted for age, sex, race, education level, marital status, the ratio of household income to poverty (PLR), alcohol consumption, smoking status, BMI, hypertension, and family history of CVD.  1–OHNAP = urinary metabolites of 1–Hydroxynaphthalene; 2–OHNAP = urinary metabolites of 2–Hydroxynaphthalene; 3–OHFLU = urinary metabolites of 3–Hydroxyfluorene; 2–OHFLU = urinary metabolites of 2–Hydroxyfluorene; 1–OHPHE = urinary metabolites of 1–Hydroxyphenanthrene; 1–OHPYR = urinary metabolites of 1–Hydroxypyrene; 2&3–OHPHE = urinary metabolites of 2&3–Hydroxyphenanthrene. | | | |
