# Supplementary material for: Association of mixed polycyclic aromatic hydrocarbons exposure with cardiovascular disease and the mediating role of inflammatory indices in US adults
Source: Environ Health Prev Med. 2024 Dec 10;29:70. doi: 10.1265/ehpm.24-00091 (PMC11652969; doi:10.1265/ehpm.24-00091)
Supplement: Supplementary file 7 — Table S5. Multivariate analysis of the association of urinary mixed of PAHs and concentration changes (95% CI) in inflammatory indicators. [file ehpm-29-070-s007.docx]

| Table S5. Multivariate analysis of the association of urinary mixed of PAHs and concentration changes (95% CI) in inflammatory indicators. | | | |
| --- | --- | --- | --- |
|  | **Inflammation index** | **Mean change (95 % CI)** | ***p* value** |
| **Mixed of PAHs** | PLR | –3.61 (–4.61, –2.60) | 0.001 |
|  | NLR | 0.42 (0.02, 0.07) | 0.001 |
|  | SII | 12.36 (3.20, 21.53) | 0.008 |
| Note: The result was adjusted as age, sex, race, education level, marital status, PLR, alcohol consumption, smoking status, BMI, hypertension, and family history of CVD.  Mixed of PAHs = Mixed of polycyclic aromatic hydrocarbons; NHANES = National Health and Nutrition Examination Survey; NLR = neutrophil–to–lymphocyte ratio; PLR = platelet–to–lymphocyte ratio; SII = systemic immunity–inflammation index. | | | |
