# Supplementary material for: Association of mixed polycyclic aromatic hydrocarbons exposure with cardiovascular disease and the mediating role of inflammatory indices in US adults
Source: Environ Health Prev Med. 2024 Dec 10;29:70. doi: 10.1265/ehpm.24-00091 (PMC11652969; doi:10.1265/ehpm.24-00091)
Supplement: Supplementary file 8 — Table S6. Association of inflammatory indicators levels with CVD (N = 9136), NHANES (2003–2016). [file ehpm-29-070-s008.docx]

| Table S6. Association of inflammatory indicators levels with CVD (N = 9136), NHANES (2003–2016). | | |  |  |
| --- | --- | --- | --- | --- |
| **Inflammation index** | **OR (95 % CI)** | ***p* value** | | |
| PLR | 0.997 (0.995, 0.999) | **0.002** | | |
| NLR | 1.273 (1.147, 1.416) | **0.000** | | |
| SII | 1.001 (1.001, 1.005) | **0.049** | | |
| Note: The result was adjusted as age, sex, race, education level, marital status, PLR, alcohol consumption, smoking status, BMI, hypertension, and family history of CVD.  CVD = cardiovascular disease; NHANES = National Health and Nutrition Examination Survey; NLR = neutrophil–to–lymphocyte ratio; PLR = platelet–to–lymphocyte ratio; SII = systemic immunity–inflammation index. | | | |  |
